# Clinical Spectrum of Monoclonal Protein and the Factors Associated with Lymphoplasmacytic Malignancies

**DOI:** 10.3390/jcm13226875

**Published:** 2024-11-15

**Authors:** Ye Hyun Kim, Yong Jun Choi, Jooheon Park, Myung Geun Shin, Eun-Hee Nah

**Affiliations:** 1 Department of Laboratory Medicine, Chonnam National University Hwasun Hospital, Hwasun 58128, Republic of Korea; yh3978@naver.com (Y.H.K.); azarsis@hanmail.net (Y.J.C.); goodjusa@naver.com (J.P.); 2 Department of Laboratory Medicine, Chonnam National University Hospital, Gwangju 61469, Republic of Korea

**Keywords:** monoclonal protein, monoclonal gammopathy of undetermined significance, lymphoplasmacytic malignancies, monoclonal gammopathy of clinical significance

## Abstract

**Background:** Monoclonal protein (MP) presents in various monoclonal gammopathies, ranging from benign conditions such as monoclonal gammopathy of undetermined significance (MGUS) to life-threatening conditions such as lymphoplasmacytic malignancies (LPMs), which include multiple myeloma (MM) and Waldenström macroglobulinemia (WM). Few studies have comprehensively assessed the clinical spectrum of MP and its factors associated with LPMs. This study aimed to determine the clinical spectrum of MP and identify factors associated with LPMs. **Methods:** This retrospective study included patients who were first tested for capillary electrophoresis (CEP) and identified as having MP between 2014 and 2023 at two university hospitals. Univariate (crude) and multivariate (adjusted) logistic regression analyses were performed to identify factors associated with LPMs. **Results:** Among the 1135 included patients with MP, 744 (65.6%) were diagnosed with LPMs and 391 (34.4%) with MGUS. Among the 391 patients with MGUS, 310 (79.3%) had at least 1 clinical association, including 204 with renal diseases, 35 with autoimmune diseases, 33 with chronic liver diseases, 22 with hematologic diseases, and 96 with other conditions. Multivariate analyses indicated that LPMs were associated with female sex (OR = 2.08), lower age (OR = 0.95), higher MP level (OR = 3.53), an abnormal FLC ratio (OR = 6.15), lower hemoglobin level (OR = 0.82), and higher total calcium level (OR = 1.81) (all *p* < 0.05). **Conclusions:** This study provides insight into the distribution of MPs and their clinical association with MGUS and identifies factors related to LPM. These can help clinicians manage patients more effectively in the early stages of these conditions.

## 1. Introduction

Monoclonal protein (MP) is a complete immunoglobulin molecule or its fragment formed by the clonal proliferation of clonal plasma cells or B lymphocytes [1]. MP is found in various monoclonal gammopathies, ranging from benign conditions such as monoclonal gammopathy of undetermined significance (MGUS) to life-threatening conditions such as lymphoplasmacytic malignancies (LPMs) that include multiple myeloma (MM), light chain amyloidosis (AL), and Waldenström macroglobulinemia (WM) [2]. Although reports on the clinical spectrum of MP exist [2,3,4], further data are needed to comprehensively assess the distribution and clinical characteristics of MP across diverse racial populations and medical settings.

The identification, quantification, and typing of MP are crucial to diagnose, classify, and monitor monoclonal gammopathies in LPMs and MGUS [5]. Protein electrophoresis (EP) followed by immunofixation electrophoresis (IFE) on serum or urine is the current laboratory method for detecting MP. Capillary EP (CEP) has emerged as a feasible alternative to conventional gel EP that has demonstrated advantages such as full automation and strong analytical performance [6]. MP testing is frequently ordered for patients with symptoms associated with LPM; however, such symptoms are generally non-specific, leading many patients with suspected LPM to eventually be diagnosed with MGUS.

MGUS was initially used to describe asymptomatic patients with detectable MP in the serum without evidence of LPMs at the time of diagnosis [7]. MGUS is a diagnosis of exclusion, which is characterized by the presence of serum MP secreted by clonal plasma cells without end-organ damage indicative of LPM. Over the last two decades, the term monoclonal gammopathy of clinical significance (MGCS) has been introduced to categorize a complex and heterogeneous group of non-malignant monoclonal gammopathies. These are classified based on the primarily involved organ or system, with kidneys, skin, nerves, and eyes being the most frequently affected [8,9]. Non-hematologists also frequently order MP tests in patients presenting with symptoms associated with LPMs. The majority of the patients suspected to have dysproteinemias either have no MP or are eventually incidentally diagnosed with MGUS. MGUS has been demonstrated to be an obligate precursor of LPMs. MGUS has also been linked to various non-malignant diseases, but a direct causal relationship has not been established in many of these cases [10,11]. These conditions may improve with plasma cell-directed therapy, making it vital for clinicians to be aware of their association with MGUS.

Once the diagnosis of MGUS is made, patients should be risk stratified in accordance with the International Myeloma Working Group (IMWG) guidelines. Bone marrow examinations and skeletal surveys are generally performed to rule out LPMs in intermediate- and high-risk patients with suspected MGUS. In contrast, low-risk patients can continue to follow up with non-hematologists. Although the rate of progression to LPM is considerably low, it is important for clinicians to remain vigilant of the red flag symptoms and laboratory abnormalities such as hemoglobin and calcium levels, and free light chain (FLC) ratios associated with progression. While specific indications for these invasive workups are based on a risk stratification model proposed by expert groups [12,13], factors associated with LPMs warrant investigation for effective follow-up testing for monoclonal gammopathy at the time of initial diagnosis. This study, therefore, aimed to determine (1) the clinical spectrum of MP identified by CEP in South Korean tertiary hospitals, (2) clinical associations with MGUS, and (3) factors associated with LPMs.

## 2. Materials and Methods

### 2.1. Study Design and Data Collection

**Figure 1:**
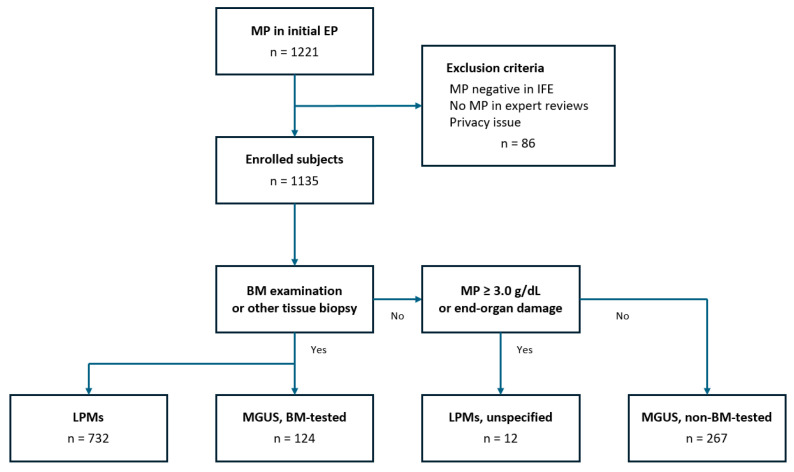
Flowchart of the study.

This cross-sectional retrospective study included patients who were first tested for CEP and identified as having MP between January 2014 and December 2023 at two university hospitals. The exclusion criteria were as follows: (i) MP negative in IFE, (ii) no MP in expert reviews, and (iii) patients who declared that they did not want their medical records to be used. Data were collected in a central database of Chonnam National University Hospital Information System. A laboratory information system was utilized to review laboratory results, and clinical data were gathered through the Clinical Data Warehouse and medical chart reviews. Clinical data were evaluated, including age, sex, and comorbidities (e.g., diabetes mellitus and hypertension) and final diagnosis. Laboratory data included CEP and serum IFE patterns (e.g., MP level and type), serum FLC, along with the ratio of kappa to lambda (κ/λ), complete blood count (CBC) parameter, total protein, albumin, creatinine, blood urea nitrogen (BUN), total and ionized calcium, ß2-microglobulin, C-reactive protein (CRP), liver transaminases, lactate dehydrogenase (LDH), N-terminal pro-B-type natriuretic peptide, and uric acid. Bone marrow (BM) tested-MGUS was diagnosed based on the IMWG diagnostic criteria [14]: (i) <3 g/dL MP in serum, (ii) <10% monoclonal plasma cells in bone marrow, and (iii) no end-organ damage (hypercalcemia, renal failure, anemia, or bone lesions). Non-BM-tested MGUS was defined based on a diagnosis of exclusion, characterized by the absence of ≥3 g/dL serum MP secreted by clonal plasma cells without end-organ damage indicative of LPM. The defined LPMs included AL, WM, chronic lymphocytic leukemia, non-Hodgkin lymphoma, and MM (Figure 1).

### 2.2. Laboratory Measurements

Venous blood was drawn after an overnight fast and centrifuged at 3000 rpm for 10 min to separate serum. CEP was performed using Capillarys 2 or Capillarys 3 OCTA (Sebia, Lisses, France), which were validated for precision and linearity based on Clinical Laboratory Standards Institute guidelines according to the manufacturer’s instruction. Serum IFE (CAPI 3 Immunotyping, Sebia) was used to determine the MP immunotyping of serum samples with an abnormal band. The serum-free light chain (FLC) assay was performed using the Freelite Kit (Binding Site, Birmingham, UK). The standard perpendicular drop method (calculating the MP content from peak to base) was employed to quantify MP using the following equation: MP concentration (g/dL) = MP (%) × total protein concentration (g/dL) [15].

### 2.3. Statistical Analysis

All statistical analyses were performed using R Statistical Software v4.3.1 (R Foundation for Statistical Computing, Vienna, Austria). Data were represented by median and interquartile ranges (IQR) or frequency (percentage) values. Numerical variables that were not normally distributed were compared between groups using the Wilcoxon rank-sum test or Kruskal–Wallis rank-sum test with post-hoc analysis using Dunn’s test with Benjamini–Hochberg adjustment. Categorical variables were compared using the chi-square test. Univariate (crude) and multivariate (adjusted) logistic regression analyses were performed to identify factors associated with LPM after adjustment for age, sex, and laboratory data. Probability values of *p* < 0.05 were considered significant.

## 3. Results

### 3.1. Characteristics of the Subjects with MP

The study involved 1135 subjects with MP diagnosed using CEP between January 2014 and December 2023: 182, 188, 192, 258, and 315 of the subjects were diagnosed during 2014–2015, 2016–2017, 2018–2019, 2020–2021, and 2022–2023, respectively. These 1135 subjects were analyzed, and 54 (4.8%) were identified with MP only in urine. Orders for MP screening initially originated most often from hematology (59.3%), followed by nephrology (16.2%), emergency medicine (10.0%), cardiology (2.6%), pulmonology (2.2%), and neurology (1.6%) (Supplementary Figure S1). The median age of the subjects was 71 years (IQR = 63–77, range = 18–94 years). The cohort comprised 664 males (58.5%) and 471 females (41.5%). The median level of serum MP was 1.84 g/dL (IQR = 0.64–3.93, range = 0.01–9.45). IgG was the most commonly affected immunoglobulin (49.3%), followed by IgA (14.6%), free FLC (6.3%), IgM (6.0%), and biclonal (1.3%). LPM and MGUS were diagnosed in 744 (65.6%) and 391 (34.4%) patients, respectively. Among the 391 cases of MGUS, 124 cases (31.7%) had bone marrow findings that met the IMWG diagnostic criteria for MGUS, with the proportion increasing from 2014 to 2023 (Table 1 and Supplementary Figure S2).

### 3.2. Demographic Characteristics and Disease Distributions of Monoclonal Gammopathy According to Immunoglobulin Isotype

**Figure 2:**
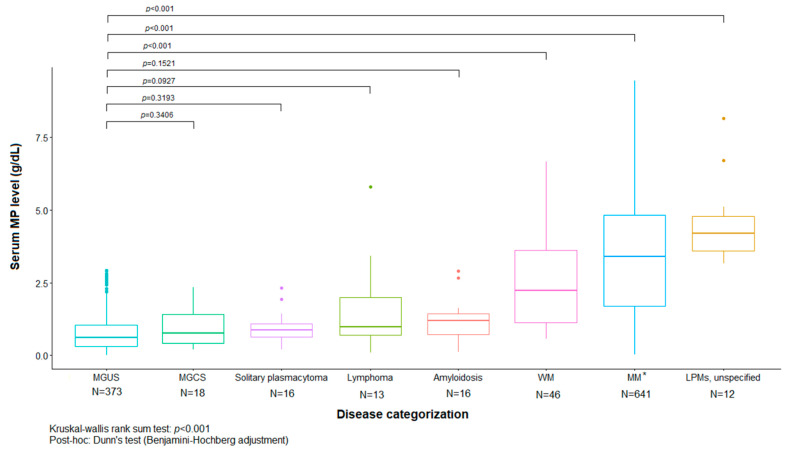
Comparison of serum monoclonal protein levels based on disease categorization (n = 1135). * Smouldering multiple myeloma and plasma cell leukemia were included. Abbreviations: MP, monoclonal protein; MGUS, monoclonal gammopathy of undetermined significance; MGCS, monoclonal gammopathy of clinical significance; WM, Waldenström’s macroglobulinemia; MM, multiple myeloma.

Among patients with MP of the IgG, IgA, and FLC isotypes, MM was the most frequently diagnosed condition, followed by MGUS. WM was the most frequently diagnosed condition in the IgM isotype group. The MP level was highest in those with MM, followed by WM and AL. The MP levels of the IgG, IgA, IgM, and FLC types were found to differ between the MM group and the MGUS group, with higher levels observed in the MM group compared with MGUS. In the comparison between MGUS and MGCS, the MP level appeared to be higher in MGCS than in MGUS, but there was no statistical significance (Table 2 and Figure 2).

### 3.3. Clinical Associations with MGUS

**Figure 3:**
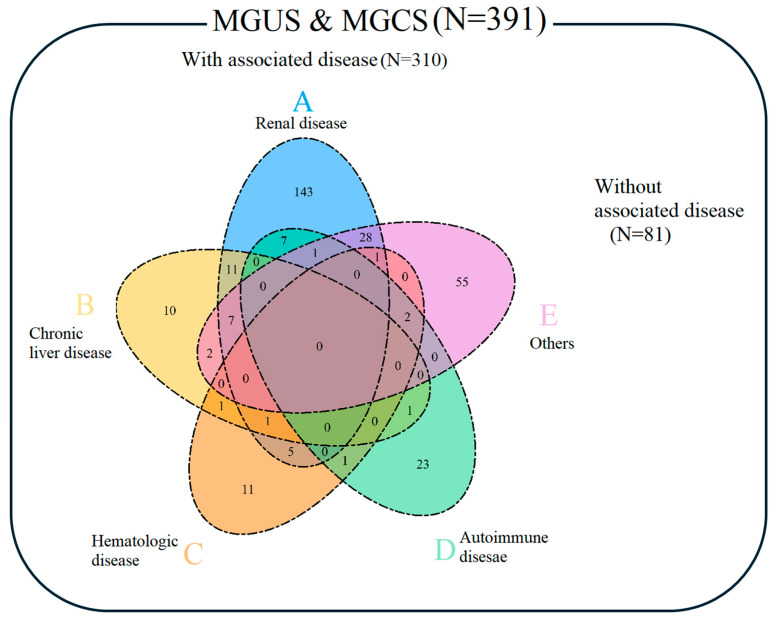
Clinical associations with monoclonal gammopathy of undetermined significance (MGUS) and monoclonal gammopathy of clinical significance (MGCS).

Of the 391 patients with MGUS and MGCS, 310 (79.3%) had at least one clinical association: 204 with renal diseases, 35 with autoimmune diseases, 33 with chronic liver diseases, 22 with hematologic diseases, and 96 with other conditions (44 with infectious diseases, 18 with solid organ malignancies, 10 with gout, and 24 with other diseases). The most common diagnoses within these categories were chronic kidney disease, infectious diseases, autoimmune diseases, chronic B/C hepatitis or liver cirrhosis, and myelodysplastic syndrome. Among the 310 cases, 68 (21.9%) had two or more clinical associations with MGUS (Supplementary Table S1 and Figure 3).

### 3.4. Comparison Between MGUS and LPMs

Compared with patients diagnosed with MGUS, those with LPM were younger and had a lower proportion of males, lower levels of creatinine, BUN, CRP, liver transaminases, LDH, and lower white blood cell (WBC) count, hemoglobin, and platelet count (all *p* < 0.05). They also exhibited an increased red cell distribution width, higher total and ionized calcium levels, higher total protein and ß2-microglobulin levels, and an abnormal FLC ratio (all *p* < 0.05) (Table 3). Among MGUS patients, BM-tested MGUS patients had higher MP levels and lower levels of creatinine, BUN, CRP, LDH, and lower WBC count compared with non-BM-tested MGUS patients (Supplementary Table S2).

### 3.5. Factors Associated with LPM in Subjects with MP

The multivariate analyses indicated that LPMs were associated with female sex (OR = 2.08, 95% CI = 1.18–3.75), lower age (OR = 0.95, 95% CI = 0.92–0.98), higher MP level (OR = 3.53, 95% CI = 2.42–5.31), an abnormal FLC ratio (OR = 6.15, 95% CI = 3.44–11.24), lower hemoglobin level (OR = 0.82, 95% CI = 0.71–0.94), and higher total calcium level (OR = 1.81, 95% CI = 1.26–2.73) (all *p* < 0.05) (Table 4).

## 4. Discussion

This study recruited patients from tertiary university hospitals to explore the clinical spectrum of MP using CEP, its clinical association with MGUS, and the factors associated with lymphoplasmacytic malignancies (LPMs). Two-thirds of the patients were diagnosed with LPMs, while MGUS represented a smaller proportion, and MGCS was rare (1.6%). MM was the most prevalent condition among those diagnosed with LPMs (81.2%), a trend that aligned with the findings for the IgG and IgA isotypes, whereas WM was more prevalent in the IgM isotype. Only about one-third of MGUS cases had bone marrow findings that met the IMWG diagnostic criteria. A clinical association with MGUS was observed in 79.3% of cases, and renal disease was the most commonly associated condition. The serum abnormal FLC κ/λ ratio had the strongest association with LPMs.

While MGUS was frequently diagnosed at the Mayo Clinic during 1960–2017 [2], two-thirds of the individuals with MP in our cohort were diagnosed with LPMs. This probably reflects the source of data for the initial MP screening, which primarily originated from the hematology department that focused on suspected LPMs, particularly MM. The higher prevalence of MM among LPMs was consistent with the findings of a previous study [2], a trend that was also observed for the IgG and IgA isotypes. However, WM was the most common in the IgM isotype. These findings were consistent with previous reports that while IgG or IgA MGUS typically progressed to MM, IgM MGUS progressed to lymphoplasmacytic lymphoma or other B-cell lymphoproliferative disorders [16,17].

MM had the highest MP level in our study, followed by WM and AL. Monoclonal gammopathies produce MP that is detectable as a discrete band on EP of serum or urine. The MP concentration may vary significantly, with a condition with low tumor burden, such as AL, producing only low concentrations compared with those observed in conditions such as MM and WM [18]. The MP level also appeared to be higher in MGCS than in MGUS in our cohort, but there was no statistical significance. This finding was inconsistent with findings from other studies in which a higher MP level was observed in the monoclonal gammopathy of renal significance (MGRS) group [19]. This discrepancy is thought to be due to the fact that the number of MGCS cases is much smaller compared with MGUS in our cohort.

MP can be detected in investigations for suspected MM, WM, or AL but also in general clinical practice [20]. MP testing can also identify non-malignant diseases associated with MGUS, which are now collectively termed MGCS [9,21]. In our study, 79.3% of MGUS patients had clinical associations, which is higher than in another study where only 21% had secondary disease associations [19]. The most common diagnoses were chronic kidney disease, infectious disease, autoimmune disease, chronic hepatitis B/C or liver cirrhosis, and myelodysplastic syndrome in each disease category. The most frequent association with MGUS in our cohort was renal disease, especially chronic kidney disease, which differed from other studies in which chronic inflammation or autoimmune diseases were more common [19,22,23,24]. This discrepancy may reflect differences in the study populations.

MGRS has been proposed to identify patients who would traditionally be categorized under MGUS but exhibit renal impairments. MGRS is linked to various renal pathologies [25] that affect renal function and increase the risks of mortality and renal graft recurrence. Immediate treatment rather than regular monitoring is recommended for patients with MGRS [26]. It is crucial to establish a causal relationship between MP and renal abnormalities when diagnosing MGRS, which necessitates kidney biopsies in suspected cases [25,27]. The prevalence of renal disease associated with MGUS was 65.8%, but that with MGRS was less than 1%, highlighting the need for greater clinician awareness and proactive diagnostic approaches such as kidney biopsies.

MP produced by small clones of plasma cells or B cells can also affect other organ systems, such as the peripheral nerves and skin. The term MGCS was introduced in 2018 with the aim of improving the classification of patients with organ damage due to MP from non-malignant plasma cells or B cells [9]. MP is found in 3–5% of patients with unexplained peripheral neuropathy of unknown etiology, therefore warranting screening for MGUS [28,29,30]. The most common indications for MP testing included neuropathy, renal disease, anemia, bone disorders or connective tissue pain, and cutaneous disease at the Mayo Clinic [31]. The subsequent diagnoses were sensory or motor neuropathy, chronic kidney disease, iron deficiency anemia, and osteoporosis/osteopenia [31]. Monoclonal gammopathy of neurological significance was presented in less than 1% of our cohort. Although the mechanisms underlying these diseases are not fully understood, increased recognition could clarify the pathogenic role of MP and lead to improved therapies. The accurate diagnosis of MGCS requires careful exclusion of coincidental associations, particularly given the high prevalence of MP in the elderly. Both clinical and histopathological factors must be considered when linking organ damage to MP.

MGUS is typically an incidental finding during evaluations for LPMs such as MM, AL, or WM. Routine baseline bone marrow examinations and skeletal radiography are not recommended for low-risk patients with MGUS despite it being a known precursor to advanced LPMs [32,33]. The factors associated with LPM in our study included female sex, lower age, higher MP and total calcium levels, lower hemoglobin level, and abnormal FLC κ/λ ratio. Sex differences in the incidence and outcomes of several cancers are well established. There is a clear sex disparity in MM incidence, which is more common in males than females. The reasons behind this are not well understood, and the impact of sex on patient outcomes is unclear. A study demonstrated fundamental differences in genetic lesions underlying the biology of MM between males and females. However, they found that progression-free survival and overall survival were the same in both sexes [34]. The LPMs in our study comprised a heterogeneous set of diagnoses. The association between LPMs and sex needs to be evaluated for each individual LPM. Previous studies have demonstrated that several laboratory tests performed at the time of diagnosis of MGUS are useful in predicting the risk of progression to MM or other related malignant conditions [12,35]. A strong predictor of progression is the serum MP concentration [35]. These results are consistent with our findings. Among the associated risk factors, the serum FLC κ/λ ratio showed the strongest association with LPMs in our study, suggesting that this ratio is a significant indicator of the risk of progression to LPMs. The FLC ratio has been recognized to predict progression to malignant conditions in patients with MGUS, independent of the concentration and type of serum MP [12]. An abnormal FLC is considered a marker of clonal expansion [36]. These associated factors can help guide the decision to perform more invasive tests, such as bone marrow examinations, in addition to serum monoclonal EP and routine laboratory tests, for early intervention and screening in patients with high-risk MP profiles.

This study has several limitations. There is a possibility that some patients classified as non-BM-tested MGUS may have LPM. The data were derived from patients at two tertiary university hospitals, which may not be fully representative of all individuals with MP detected in the serum or urine. Additionally, the reliance on hospital information systems and medical records could have introduced coding errors. Given that this study uses data from the past 10 years, it is also subject to limitations such as potential selection bias, missing data, and unmeasured confounders. The retrospective, cross-sectional design further restricts the ability to draw causal inferences. Despite these limitations, the use of a dataset spanning 10 years allowed for analyses of a substantial cohort of patients with MP.

## 5. Conclusions

This study highlights the disease distribution of MP, conditions associated with MGUS, and factors related to LPMs by integrating various clinical data. Understanding the clinical associations with MGUS and the spectrum of MP-related diseases can improve patient management and facilitate earlier interventions. Additionally, awareness of the factors associated with LPMs could help identify patients at risk of progression to more advanced LPMs early in the disease course, enabling preemptive intervention before organ damage occurs. Future directions will focus on further refining our understanding of these findings through molecular and genetic signatures.

## Figures and Tables

**Table 1:** Characteristics of the study subjects with monoclonal protein (MP).

Characteristics	n = 1135
Age (years)	71 (18–94)
Sex	
Male	664 (58.5%)
Female	471 (41.5%)
Serum MP level (g/dL)	1.84 (0.01–9.45)
Immunotyping distribution in serum IFE	
IgG-κ/IgG-λ	337 (29.7%)/222 (19.6%)
IgA-κ/IgA-λ	95 (8.4%)/70 (6.2%)
IgM-κ/IgM-λ	56 (4.9%)/12 (1.1%)
Free κ/Free λ	19 (1.7%)/52 (4.6%)
Biclonal	15 (1.3%)
Technical error	2 (0.2%)
Only in urine	54 (4.8%)
Not tested	201 (17.7%)
Disease categorization	
Lymphoplasmacytic malignancy (LPM)	744 (65.6%)
Multiple myeloma	604 (53.2%)
Smouldering multiple myeloma	23 (2.0%)
Plasma cell leukemia	14 (1.2%)
Waldenström’s macroglobulinemia	46 (4.1%)
Solitary plasmacytoma	16 (1.4%)
Amyloidosis	16 (1.4%)
Mature lymphoid neoplasm	13 (1.1%)
LPM, unspecified	12 (1.1%)
MGUS *	391 (34.4%)
MGUS, BM-tested	124 (10.9%)
MGUS, non-BM-tested	267 (23.5%)

Data are n (%) or median (range) values. * Monoclonal gammopathy of renal significance (n = 8) and monoclonal gammopathy of neurological significance (n = 10) patients were included. Abbreviations: IFE, immunofixation electrophoresis; MGUS, monoclonal gammopathy of undetermined significance; BM, bone marrow.

**Table 2:** Demographic characteristics and disease distributions of monoclonal gammopathies in each immunoglobulin isotype.

	Disease Categorization	n = 863	Type (κ/λ)	Sex (M/F)	Age (Years)	MP Level (g/dL)
IgG	Total	559 (100.0)	337/222	319/240	71 (31–94)	2.59 (0.03–9.45)
	Multiple myeloma *	369 (66.0)	228/141	190/179	70 (37–93)	3.70 (0.13–9.45)
	Solitary plasmacytoma	13 (2.3)	7/6	9/4	60 (46–76)	0.95 (0.19–2.31)
	Amyloidosis	6 (1.1)	3/3	4/2	71 (31–76)	1.46 (0.90–2.90)
	Mature lymphoid neoplasm	3 (0.5)	3/0	2/1	59 (58–68)	1.05 (0.09–1.09)
	LPM, unspecified	4 (0.7)	1/3	3/1	79 (49–82)	5.30 (3.24–8.17)
	MGCS	12 (2.2)	3/9	11/1	71.5 (62–85)	0.86 (0.20–2.35)
	MGUS	152 (27.2)	92/60	100/52	73 (32–94)	0.73 (0.03–2.93)
IgA	Total	165 (100.0)	95/70	90/75	70 (47–86)	2.74 (0.01–8.84)
	Multiple myeloma *	126 (76.4)	78/48	66/60	70 (49–86)	3.49 (0.24–8.84)
	Amyloidosis	4 (2.4)	1/3	2/2	74.5 (66–78)	1.18 (0.11–1.43)
	LPM, unspecified	1 (0.6)	0/1	0/1	76 (76–76)	4.35 (4.35–4.35)
	MGCS	1 (0.6)	1/0	1/0	57 (57–57)	1.43 (1.43–1.43)
	MGUS	33 (20.0)	15/18	21/12	69 (47–85)	0.71 (0.01–1.42)
IgM	Total	68 (100.0)	56/12	50/18	71.5 (42–90)	1.20 (0.02–6.67)
	Multiple myeloma *	2 (2.9)	2/0	2/0	70.5 (64–77)	3.84 (1.07–6.60)
	WM	41 (60.3)	38/3	33/8	70 (42–88)	2.26 (0.57–6.67)
	Amyloidosis	1 (1.5)	1/0	1/0	85 (85–85)	0.41 (0.41–0.41)
	Mature lymphoid neoplasm	4 (5.9)	4/0	1/3	70 (64–75)	0.76 (0.40–5.80)
	LPM, unspecified	1 (1.5)	0/1	1/0	83 (83–83)	4.58 (4.58–4.58)
	MGCS	2 (2.9)	1/1	1/1	75 (74–76)	0.34 (0.30–0.38)
	MGUS	17 (25.0)	10/7	11/6	72 (58–90)	0.29 (0.02–1.26)
FLC	Total	71 (100.0)	19/52	43/28	68 (33–89)	0.40 (0.02–6.54)
	Multiple myeloma *	63 (88.7)	14/49	36/27	70 (33–89)	0.43 (0.03–6.54)
	Solitary plasmacytoma	1 (1.4)	1/0	0/1	57 (57–57)	0.20 (0.20–0.20)
	MGUS	7 (9.9)	4/3	7/0	65 (50–82)	0.27 (0.02–0.88)

Data are n (%), n/n, or median (range) values. * Smouldering multiple myeloma and plasma cell leukemia were included. Abbreviations: MP, monoclonal protein; LPM, lymphoplasmacytic malignancy; MGCS, monoclonal gammopathy of clinical significance; MGUS, monoclonal gammopathy of undetermined significance; WM, Waldenström’s macroglobulinemia; FLC, free light chain.

**Table 3:** Comparison between lymphoplasmacytic malignancies (LPMs) and MGUS.

Characteristic	Overall (n = 1135)	LPMs (n = 744)	MGUS (n = 391)	*p* *
Sex				<0.001
Male	664 (59%)	404 (54%)	260 (66%)	
Female	471 (41%)	340 (46%)	131 (34%)	
Age (years)	71 (63–77)	70 (63–76)	72 (64–79)	<0.001
Serum MP level (g/dL)	1.84 (0.64–3.93)	3.22 (1.41–4.69)	0.60 (0.32–1.08)	<0.001
Serum IFE types				
IgG	559 (49.3%)	395 (53.1%)	164 (42%)	
IgA	165 (14.5%)	131 (17.6%)	34 (8.7%)	
IgM	68 (6.0%)	49 (6.6%)	19 (4.8%)	
FLC	71 (6.3%)	64 (8.6%)	7 (1.8%)	
Biclonal	15 (1.3%)	11 (1.5%)	4 (1.0%)	
Not obtained	257 (22.6%)	94 (12.6%)	163 (41.7%)	
FLC_kappa (mg/L)	57 (18–234)	55 (14–377)	59 (29–136)	0.8
FLC_lambda (mg/L)	30 (11–160)	18 (8–319)	46 (23–120)	<0.001
FLC ratio				<0.001
Normal	206 (21%)	58 (8.6%)	148 (51%)	
Abnormal	760 (79%)	620 (91%)	140 (49%)	
WBC (×10^3^/μL)	6.0 (4.7–7.6)	5.6 (4.4–7.2)	6.7 (5.6–8.8)	<0.001
RBC (×10^6^/μL)	3.23 (2.75–3.78)	3.11 (2.68–3.64)	3.48 (3.01–4.13)	<0.001
Hemoglobin (g/dL)	10.1 (8.7–11.8)	9.7 (8.4–11.4)	10.8 (9.5–12.8)	<0.001
RDW (%)	14.4 (13.3–16.1)	14.8 (13.5–16.5)	13.9 (13.0–15.2)	<0.001
Platelet (×10^3^/μL)	201 (151–254)	194 (143–251)	209 (170–272)	<0.001
Lymphocyte (×10^3^/μL)	1.70 (1.23–2.20)	1.80 (1.28–2.27)	1.54 (1.09–2.02)	<0.001
Monocyte (×10^3^/μL)	0.47 (0.34–0.64)	0.45 (0.33–0.61)	0.53 (0.39–0.71)	<0.001
Neutrophil (×10^3^/μL)	3.40 (2.36–4.85)	3.07 (2.17–4.43)	4.08 (3.05–6.20)	<0.001
Reticulocyte count (%)	1.43 (1.05–2.11)	1.39 (1.00–2.02)	1.59 (1.14–2.26)	0.024
Total protein (g/dL)	7.8 (6.7–9.6)	8.9 (7.3–10.2)	6.9 (6.2–7.5)	<0.001
Albumin (g/dL)	3.5 (3.0–4.0)	3.5 (3.1–4.0)	3.6 (2.9–4.2)	0.2
Creatinine (mg/dL)	1.08 (0.80–1.70)	1.04 (0.80–1.50)	1.20 (0.83–2.10)	<0.001
BUN (mg/dL)	20 (14–32)	19 (14–28)	22 (15–42)	<0.001
Total calcium (mg/dL)	9.0 (8.5–9.6)	9.15 (8.7–9.8)	8.9 (8.2–9.4)	<0.001
Ionized calcium (mEq/L)	2.46 (2.38–2.54)	2.46 (2.38–2.56)	2.44 (2.34–2.50)	0.002
ß2-microglobulin (μg/L)	4374 (2801–7886)	4675 (2988–8384)	3434 (2283–6470)	<0.001
CRP (mg/dL)	0.3 (0.1–1.3)	0.3 (0.1–1.0)	0.5 (0.1–3.6)	0.002
AST (U/L)	23 (17–31)	22 (17–29)	26 (19–34)	<0.001
ALT (U/L)	17 (11–24)	16 (11–23)	18 (12–29)	0.005
LDH (U/L)	390 (318–491)	375 (301–474)	421 (353–528)	<0.001
NT-proBNP (pg/mL)	309 (122–1130)	309 (126–970)	355 (95–2306)	0.8
Uric acid (mg/dL)	6.2 (5.0–7.8)	6.3 (5.0–7.8)	6.2 (5.0–7.7)	0.7
Hypertension				<0.001
No	548 (48%)	387 (52%)	161 (41%)	
Yes	587 (52%)	357 (48%)	230 (59%)	
Diabetes mellitus				<0.001
No	829 (73%)	569 (76%)	260 (66%)	
Yes	306 (27%)	175 (24%)	131 (34%)	

Data are n (%) or median (interquartile range) values. * Pearson’s Chi-squared test; Wilcoxon rank sum test. Abbreviations: MGUS, monoclonal gammopathy of undetermined significance; MP, monoclonal protein; IFE, immunofixation electrophoresis; FLC, free light chain; WBC, white blood cell; RBC, red blood cell; RDW, red cell distribution width; BUN, blood urea nitrogen; CRP, C-reactive protein; AST, aspartate transaminase; ALT, alanine transaminase; LDH, lactate dehydrogenase; NT-proBNP, N-terminal pro-B-type natriuretic peptide.

**Table 4:** Factors associated with lymphoplasmacytic malignancies in multivariate logistic regression analysis (n = 640) *.

Characteristic	Univariate Analyses			Multivariate Analyses		
	OR	95% CI	*p*	OR	95% CI	*p*
Sex (reference: male)	1.87	1.26–2.80	0.002	2.08	1.18–3.75	0.013
Age (years)	0.98	0.96–1.00	0.025	0.95	0.92–0.98	0.001
Serum MP level (g/dL)	3.07	2.47–3.91	<0.001	3.53	2.42–5.31	<0.001
Abnormal FLC ratio	12.3	7.81–19.50	<0.001	6.15	3.44–11.24	<0.001
WBC (×10^3^/μL)	0.94	0.90–0.99	0.015	0.50	0.23–1.16	0.104
Hemoglobin (g/dL)	0.73	0.67–0.79	<0.001	0.82	0.71–0.94	0.005
RDW (%)	1.31	1.18–1.47	<0.001			
Platelet (×10^3^/μL)	0.82	0.67–0.99	0.038			
Lymphocyte (×10^3^/μL)	1.29	1.03–1.67	0.045	2.53	0.90–7.09	0.080
Monocyte (×10^3^/μL)	0.79	0.51–1.25	0.3			
Neutrophil (×10^3^/μL)	0.90	0.84–0.96	0.004	2.06	0.86–4.80	0.104
Total protein (g/dL)	2.02	1.76–2.35	<0.001	0.80	0.59–1.09	0.148
Albumin (g/dL)	0.64	0.48–0.85	0.002			
Creatinine (mg/dL)	0.98	0.87–1.11	0.7			
Total calcium (mg/dL)	1.59	1.27–2.02	<0.001	1.81	1.26–2.73	0.003
ß2-microglobulin (μg/L)	1.06	1.02–1.11	0.003	1.04	1.00–1.09	0.095
LDH (U/L)	0.93	0.85–1.02	0.12			
Hypertension	0.68	0.47–1.00	0.051			
Diabetes mellitus	0.65	0.43–0.99	0.04			

* Lymphoplasmacytic malignancies (n = 503), monoclonal gammopathy of undetermined significance (n = 137). Abbreviations: MP, monoclonal protein; LDH, lactate dehydrogenase; WBC, white blood cell; RDW, red cell distribution width; FLC, free light chain; OR, odds ratio; CI, confidence interval.

## Data Availability

The data presented in this study are available on request from the corresponding author.

## Supplementary Materials

The following supporting information can be downloaded at: https://www.mdpi.com/article/10.3390/jcm13226875/s1, Figure S1: Distribution of medical specialties requesting capillary electrophoresis; Figure S2: The changes in monoclonal protein distribution from 2014 to 2023; Table S1: Clinical associations with monoclonal gammopathy of undetermined significance by disease category; Table S2: Comparative analysis of MGUS patients who underwent bone marrow examination and those who did not.
