# Supplementary material for: Clinical Spectrum of Monoclonal Protein and the Factors Associated with Lymphoplasmacytic Malignancies
Source: J Clin Med. 2024 Nov 15;13(22):6875. doi: 10.3390/jcm13226875 (PMC11595081; doi:10.3390/jcm13226875)
Supplement: Supplementary file 1 [file jcm-13-06875-s001.zip › jcm-3297071-supplementary.pdf]

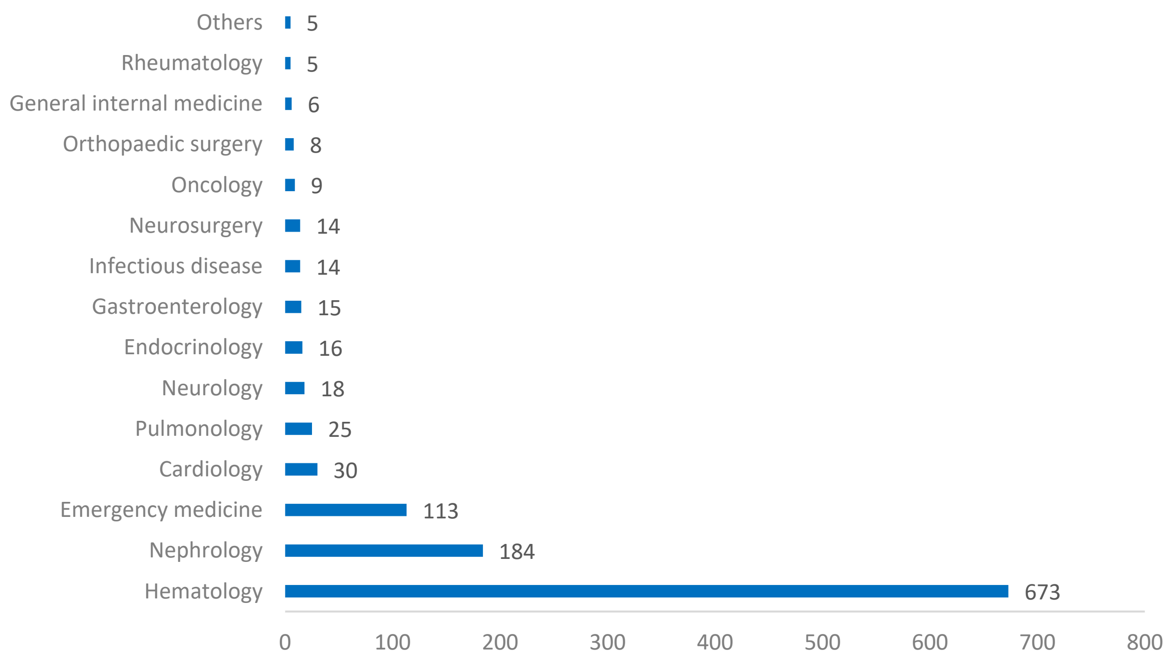

Figure S1. Distribution of medical specialties requesting capillary electrophoresis (n = 1135).

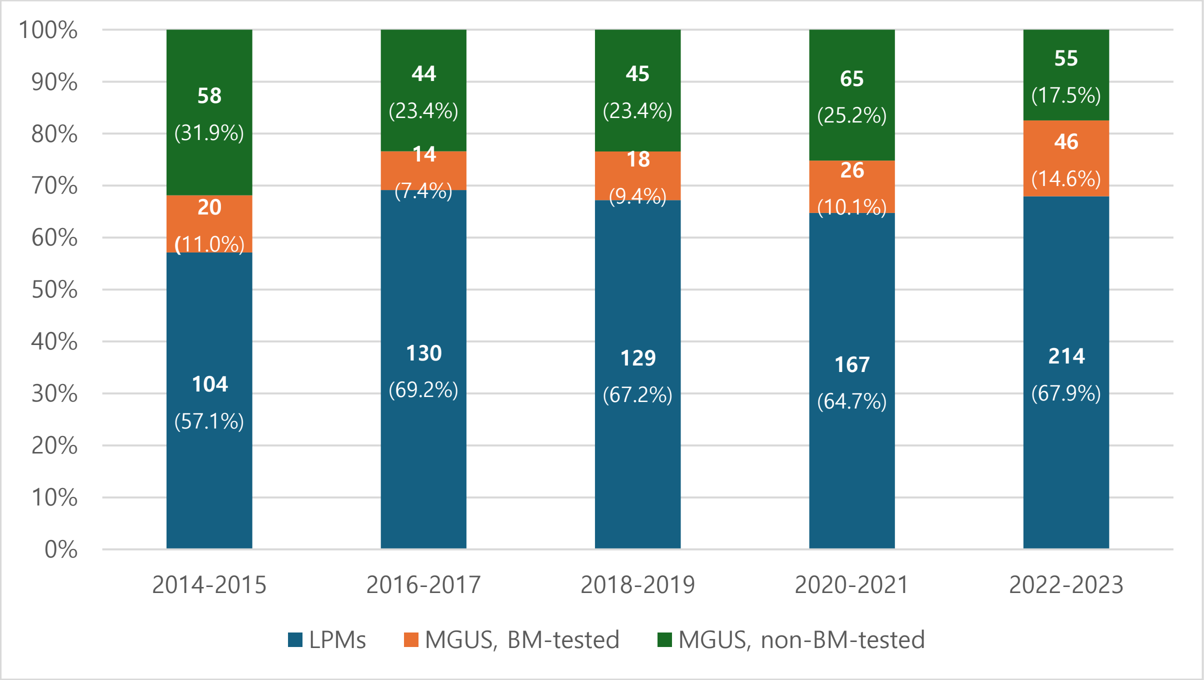

Figure S2. The changes in monoclonal protein distribution from 2014 to 2023 (n=1135). Abbreviations: LPMs, lymphoplasmacytic malignancies; MGUS, monoclonal gammopathy of undetermined significance; BM, bone marrow.

Table S1. Clinical associations with monoclonal gammopathy of undetermined significance by disease category.

**Disease category**	**n = 310**
**Renal disease**	n = 204 (65.8%)
CKD	113
AKI on CKD	34
AKI	20
Renal amyloidosis	7
Unspecified nephrotic/nephritic syndrome	7
Focal segmental glomerular sclerosis	5
IgA nephropathy	4
Minimal change disease	2
Membranous glomerulonephritis	2
Others	10
**Autoimmune disease** *	n = 35 (11.3%)
Asthma	4
Chronic inflammatory demyelinating polyneuropathy	3
Guillain-Barre syndrome	2
Multiple sclerosis	2
Myasthenia gravis	2
IgG4-related disease	2
Ankylosing spondylitis	2
Rheumatoid arthritis	2
Systemic lupus erythematosus	2
Sjogren's syndrome	2
Others	12
**Chronic liver disease**	n = 33 (10.6%)
Chronic hepatitis B or LC(B)	15
Chronic hepatitis C or LC(C)	4
LC(A)	3
LC(A+B) or LC(A+C)	3
Autoimmune hepatitis	1
Nonalcoholic fatty liver disease	1
Other LCs	6
**Other hematologic disease**	n = 22 (7.1%)
Myelodysplastic syndrome	8
Autoimmune hemolytic anemia / Cold agglutinin disease	3
Essential thrombocythemia	2
Chronic Myeloid Leukemia	2
Hereditary spherocytosis	2
Acute myeloid leukemia	1
Chronic myelomonocytic leukemia	1
Others	3
**Others**	n = 96 (31.0%)
Infectious diseases	44
Solid organ malignancies	18
Gout	10
Post-solid organ transplantation	7
Osteoporosis	4
Iron deficiency anemia	3
Benign tumors	2
Others	8

* Except for autoimmune hepatitis. Abbreviations: AKI, acute kidney injury; CKD, chronic kidney disease; LC, liver cirrhosis.

Table S2. Comparative analysis of MGUS patients who underwent bone marrow (BM) examination and those who did not.

**Characteristic**	**Overall (n = 391)**	**MGUS, BM-tested (n = 124)**	**MGUS, non-BM-tested (n = 267)**	***p*** *
Sex				0.6
Male	260 (66%)	85 (69%)	175 (66%)	
Female	131 (34%)	39 (31%)	92 (34%)	
Age (years)	72 (64–79)	72 (66–77)	73 (64–80)	0.078
Serum MP level (g/dL)	0.60 (0.32–1.08)	0.88 (0.44–1.28)	0.55 (0.30–0.93)	<0.001
Serum IFE types				
IgG	164 (42%)	77 (62.1%)	87 (32.6%)	
IgA	34 (8.7%)	19 (15.3%)	15 (5.6%)	
IgM	19 (4.8%)	9 (7.3%)	10 (3.8%)	
FLC	7 (1.8%)	1 (0.8%)	6 (2.2%)	
Biclonal	4 (1.0%)	1 (0.8%)	3 (1.1%)	
Not obtained	163 (41.7%)	17 (13.7%)	146 (54.7%)	
FLC_kappa (mg/L)	59 (29–136)	55 (22–152)	59 (32–116)	0.9
FLC_lambda (mg/L)	46 (23–120)	44 (22–115)	47 (24–125)	0.4
FLC ratio				0.021
Normal	148 (51%)	49 (43%)	99 (57%)	
Abnormal	140 (49%)	65 (57%)	75 (43%)	
WBC ($\times 10^3$/μL)	6.7 (5.6–8.8)	6.3 (4.9–8.0)	6.9 (5.7–9.2)	<0.001
RBC ($\times 10^6$/μL)	3.48 (3.01–4.13)	3.58 (3.02–4.19)	3.46 (3.01–4.13)	0.5
Hemoglobin (g/dL)	10.8 (9.5–12.8)	11.2 (9.9–13.4)	10.5 (9.2–12.6)	0.045
RDW (%)	13.9 (13.0–15.2)	13.65 (12.9–14.6)	14.1 (13.1–15.5)	0.039
Platelet ($\times 10^3$/μL)	209 (170–272)	205 (168–260)	215 (171–278)	0.5
Lymphocyte ($\times 10^3$/μL)	1.54 (1.09–2.02)	1.53 (1.10–1.95)	1.57 (1.07–2.04)	0.7
Monocyte ($\times 10^3$/μL)	0.53 (0.39–0.71)	0.50 (0.33–0.65)	0.54 (0.40–0.74)	0.01
Neutrophil ($\times 10^3$/μL)	4.08 (3.05–6.20)	3.60 (2.70–5.10)	4.40 (3.40–6.80)	<0.001
Reticulocyte count (%)	1.59 (1.14–2.26)	1.63 (1.12–2.21)	1.53 (1.14–2.26)	>0.9
Total protein (g/dL)	6.9 (6.2–7.5)	7.0 (6.2–7.7)	6.8 (6.2–7.4)	0.047
Albumin (g/dL)	3.6 (2.9–4.2)	3.8 (3.1–4.3)	3.5 (2.8–4.2)	0.031
Creatinine (mg/dL)	1.20 (0.83–2.10)	1.00 (0.80–1.44)	1.35 (0.88–2.50)	<0.001
BUN (mg/dL)	22 (15–42)	19 (14–31)	24 (16–46)	0.003
Total calcium (mg/dL)	8.9 (8.2–9.4)	9.0 (8.4–9.35)	8.8 (8.1–9.4)	0.2
Ionized calcium (mEq/L)	2.44 (2.34–2.50)	2.46 (2.36–2.50)	2.40 (2.32–2.48)	0.3
ß2-microglobulin (μg/L)	3,434 (2,283–6,470)	3,188 (2,043–5,152)	4,071 (2,477–7,307)	0.02
CRP (mg/dL)	0.5 (0.1–3.6)	0.2 (0.1–0.8)	0.7 (0.1–5.6)	0.003
AST (U/L)	26 (19–34)	23 (18–30)	26 (19–35)	0.033
ALT (U/L)	18 (12–29)	17 (11–24)	19 (12–32)	0.2
LDH (U/L)	421 (353–528)	394 (341–497)	441 (367–538)	0.009
NT-proBNP (pg/mL)	355 (95–2,306)	247 (70–682)	506 (139–4,383)	0.094
Uric acid (mg/dL)	6.2 (5.0–7.7)	6.2 (5.0–7.5)	6.2 (4.9–7.8)	0.8
Hypertension				>0.9
No	161 (41%)	51 (41%)	110 (41%)	
Yes	230 (59%)	73 (59%)	157 (59%)	
Diabetes mellitus				0.4
No	260 (66%)	86 (69%)	174 (65%)	
Yes	131 (34%)	38 (31%)	93 (35%)	

Data are n (%) or median (interquartile range) values. * Pearson's Chi-squared test; Wilcoxon rank sum test. Abbreviations: MGUS, monoclonal gammopathy of undetermined significance; MP, monoclonal protein; IFE, immunofixation electrophoresis; FLC, free light chain; AST, aspartate aminotransferase; ALT, alanine transaminase; LDH, lactate dehydrogenase; BUN, blood urea nitrogen; CRP, C-reactive protein; WBC, white blood cell; RBC, red blood cell; RDW, red cell distribution width; NT-proBNP, N-terminal pro-B type natriuretic peptide.
